# Prediction of Protein Subcellular Localization Based on Fusion of Multi-view Features

**DOI:** 10.3390/molecules24050919

**Published:** 2019-03-06

**Authors:** Bo Li, Lijun Cai, Bo Liao, Xiangzheng Fu, Pingping Bing, Jialiang Yang

**Affiliations:** 1 College of Information Science and Engineering, Hunan University, Changsha 410082, China; hn.libo@163.com (B.L.); excelsior511@126.com (X.F.); 2 School of Mathematics and Statistics, Hainan Normal University, Haikou 570100, China; 3 Academics Working Station, Changsha Medical University, Changsha 410219, China; bpping@163.com

**Keywords:** protein subcellular localization, protein primary sequence, generalized chaos game representation, statistical method, support vector machine, unitary distance

## Abstract

The prediction of protein subcellular localization is critical for inferring protein functions, gene regulations and protein-protein interactions. With the advances of high-throughput sequencing technologies and proteomic methods, the protein sequences of numerous yeasts have become publicly available, which enables us to computationally predict yeast protein subcellular localization. However, widely-used protein sequence representation techniques, such as amino acid composition and the Chou’s pseudo amino acid composition (PseAAC), are difficult in extracting adequate information about the interactions between residues and position distribution of each residue. Therefore, it is still urgent to develop novel sequence representations. In this study, we have presented two novel protein sequence representation techniques including Generalized Chaos Game Representation (GCGR) based on the frequency and distributions of the residues in the protein primary sequence, and novel statistics and information theory (NSI) reflecting local position information of the sequence. In the GCGR + NSI representation, a protein primary sequence is simply represented by a 5-dimensional feature vector, while other popular methods like PseAAC and dipeptide adopt features of more than hundreds of dimensions. In practice, the feature representation is highly efficient in predicting protein subcellular localization. Even without using machine learning-based classifiers, a simple model based on the feature vector can achieve prediction accuracies of 0.8825 and 0.7736 respectively for the CL317 and ZW225 datasets. To further evaluate the effectiveness of the proposed encoding schemes, we introduce a multi-view features-based method to combine the two above-mentioned features with other well-known features including PseAAC and dipeptide composition, and use support vector machine as the classifier to predict protein subcellular localization. This novel model achieves prediction accuracies of 0.927 and 0.871 respectively for the CL317 and ZW225 datasets, better than other existing methods in the jackknife tests. The results suggest that the GCGR and NSI features are useful complements to popular protein sequence representations in predicting yeast protein subcellular localization. Finally, we validate a few newly predicted protein subcellular localizations by evidences from some published articles in authority journals and books.

## 1. Introduction

Assigning subcellular localizations for a protein is a significant step to elucidate its interaction partners, functions and potential roles in the cellular machinery [1,2]. However, experimental methods to determine subcellular localization usually involve immunolabelling or tagging, which could be laborious and time-consuming [1,3,4,5]. With the development of high-throughput genomic and proteomic sequencing techniques, there have been increasing number of protein sequences sequenced and cataloged in the protein data banks. So there is an urgent need for effective and efficient computational methods to predict protein subcellular localizations, especially for species like yeast.

Typical computational methods to predict protein subcellular localizations consist of two steps including: (1) protein sequence representation, in which each primary protein sequence was transformed into a numerical feature vector; and (2) protein classification, in which a classification model was then trained based on the feature vectors and labels of the training samples. Currently, there are generally three categories of sequence representation methods: (1) the amino acids composition based-methods, which calculate the occurrence frequencies of the 20 amino acids, but ignore the sequence-order information of each residue; (2) the Chou’s Pseudo Amino Acid Composition (PseAAC)-based methods [6,7], which not only model the amino acid composition information but also incorporate the interactions among adjacent residues. The Chou’s PseAAC based-methods achieved about an increase of 20 percent of predicting accuracy than amino acids composition-based methods; (3) the hybrid methods allowing for integrating features from multiple views, which usually increase prediction accuracy [8,9,10]. After the sequence feature was constructed, various classifiers including covariant discriminant (CDC) [10,11], nearest neighbor (NN) [12,13], support vector machine (SVM) [14], deep learning [15] and ensemble classifier [16,17] were adopted to predict protein subcellular localization.

During the past decades, significant progresses have been made on developing efficient protein sequence representations and subsequent classifiers. For example, Zhang et al. introduced several amino acid hydrophobic patterns and average power-spectral density to define a modified PseAAC. Based on these features, they predicted protein subcellular localization by employing the covariant discriminant predictor [3]. Liao et al. attempted to identify protein subcellular locations based on amino acid composition components and adjacent triune residues [6]. Chen et al. utilized the measure of diversity and increment of diversity on protein primary sequences [18]. Ding et al. represented the apoptosis protein sequences by a novel approximate entropy (ApEn)-based PseAAC and employed an ensemble classifier model as the prediction engine, of which the basic classifier is the fuzzy K-nearest neighbor [16]. Lin et al. refined the PseAAC based on the physico-chemical characteristics of the 20 amino acids, and adopted SVM to predict protein subcellular locations [19]. Zhang et al. introduced the concept of distance frequency to capture the positional distribution information of amino acids and also adopted SVM to classify proteins [2]. More recently, Yu et al. implemented the CELLO2GO (http://cello.life.nctu.edu.tw/cello2go/) web-based system server for providing protein subcellular location prediction service based on functional Gene Ontology Annotation [20]. Wan et al. introduced a multi-label subcellular-localization predictor named HybridGO-Loc that leverages not only the GO term occurrences but also the inter-term relationships [21]. Dehzangi et al. proposed two segmentation-based feature extraction methods to explore potential local evolutionary-based information for Gram-positive and Gram-negative subcellular localizations [22]. Finally, Shao et al. employed a deep model-based descriptor (DMD) to extract high-level features from protein images, which was proven to be useful for determining the subcellular localization of proteins [23]. However, due to the limitations of feature representation schemes and the relative low accuracy of classification algorithms, most current algorithms still cannot be widely employed in real applications.

To address this problem, we first introduced two novel feature representations based on Generalized Chaos Game Representation (GCGR) and novel statistics and information theory (NSI), respectively. Using the two types of features, we developed a predicting model based on unitary distances. Our experiments indicate that the model can quickly and accurately predict the subcellular localizations for yeast even without classifiers. To further evaluate the effectiveness of the proposed new features, we proposed a multi-view feature by combining these features with well-known features like PseAAC and dipeptide composition, which was fed into a SVM classification system. We then tested the performance of the proposed features and models on two yeast benchmark datasets and compared them with a few popular methods using the jackknife test. 

## 2. Results and Discussions

**Figure 1:**
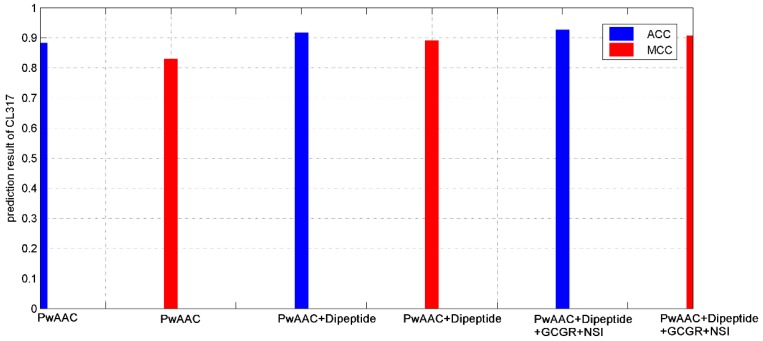
The prediction results based on CL317 using the support vector machine algorithm with different combination of features.

We listed in Table 1 and Table 2 the predicting results of the proposed model and other existing models for the jackknife test on CL317 and ZW225 respectively. As can be seen, our model achieved overall prediction accuracies of 0.8825 and 0.7736 respectively on CL317 and ZW225. The performance on CL317 outperforms some existing methods, such as Wei et al. [15] (with accuracy 0.827) and Zhang et al. [24] (with accuracy 0.88). The improvement is important considering that we only used a 2-D GCGR feature and a 3-D NSI feature, while other methods combined features like amino acid composition of 20-D and dipeptide of 400-D. We further tested the performance of combining GCGR and NSI with other widely-recognized features including pseudo-amino acid composition (PwAAC) and dipeptide composition (Dipeptide). Specifically, we applied three models including: (1) PwAAC alone, (2) fusion of features PwAAC and Dipeptide, and (3) fusion of features PwAAC, Dipeptide, GCGR and NSI into the CL317 dataset. Their prediction results for the jackknife test were summarized in Figure 1.

As Figure 1 depicts, the model combined all features achieved much higher prediction accuracy than others, indicating that: (1) feature fusion techniques are promising to improve the prediction accuracy since single-view feature can only reflect part of the information of a protein sequence; (2) the two features GCGR and NSI can be served as a helpful complementary to features like PwAAC and Dipeptide, revealing the effectiveness of the two novel feature representation techniques as well.

To further evaluate the efficiency of the feature fusion technique and improve protein subcellular location prediction accuracy, we introduced the final multiple-views based model, in which the feature vector for each protein was represented by concatenating numerical vectors from GCGR, NSI, PwAAC and Dipeptide. In addition, SVM was selected as the classifier. Comparison with other existing models using the jackknife test on CL317 and ZW225 were shown in Table 3 and Table 4, respectively.

As can be seen, our integration model achieved the highest overall accuracies, that is, 0.921 and 0.889 on CL317 and ZW225, respectively. There are two indications: (1) our proposed protein sequence feature representations including GCGR and NSI both contain some valuable information such as concentrated local information, which were not covered by previous features; (2) The integration of multiple informative features may improve prediction performance. In addition, our model achieved the highest MCCs for most of subcellular location classes on CL317 except for Cy and Me. Moreover, the MCCs and the Accs of the class Nu and Mi for our model are much better than other existing methods on ZW225.

Finally, we searched authoritative journals and publications for further validation of the predicted subcellular location of some proteins, and found that some of them have already been validated by experiments. For example, we predicted that the protein YHR196W belongs to nucleolar, which have been reported by more than 20 publications such as Eswara et al. [26] and Polymenis et al. [27]. We also predicted Sec17p to be localized in cytoplasm and the endoplasmic reticulum, consistent with Aouida et al. [28]. Thus, our model is effective in screening out potential protein subcellular locations for further experimental validation.

To summarize: first, the new simple unitary distance-based method is comparable to many methods in prediction accuracy; second, the proposed new perspectives (GCGR and NSI) truly contain some valuable information from protein primary sequence, and can be served as a complement to the existing feature representations; third, the multi-feature based model can improve the prediction accuracy notably, thus can be used to help biologist determine protein subcellular location.

However, we are fully aware that there are several limitations in this study. First of all, we only used the average of the x- and y-axis of the points in the GCGR plot, which may retrieve only partial information of the plot. An immediate option is to try other statistics of the GCGR plot such as median and percentiles. Second, the biological interpretation under the effectiveness of the features is not fully clear. Third, the current version of the software is not very user-friendly. In the future, we will devote to offer an online web service such that more biologists can use the software. We will also try to use some parallel algorithms for dealing with large scale eukaryote species including human data.

## 3. Materials and Methods

### 3.1. Datasets

In the paper, two yeast datasets CL317 and ZW225 are used for comparing different predicting models. The CL317 dataset was collected by Chen and Li [18]. The original 846 proteins explicitly annotated to one subcellular were derived from SWISSPROT (version 49.0) by European Bioinformatics Institute, Hinxton Cambridge, United Kingdom (www.ebi.ac.uk/ swissprot) [25]. Since short sequences are more like to be homologous and it is also difficult to extract enough information from them, we removed the proteins with less than 80 residues similar to Chen and Li [18]. The remaining dataset contains 317 apoptosis proteins belonging to six subcellular locations including cytoplasmic (Cy), membrane (Me), nuclear (Nu), endoplasmic reticulum (En), mitochondrial (Mi) and secreted (Se) with 112, 55, 52, 47, 34 and 17 proteins, respectively. ZW225 was curated by Zhang and Wang [24], including 225 proteins in four subcellular locations with 89 membrane proteins, 70 cytoplasmic proteins, 41 nuclear proteins and 25 mitochondrial proteins. The proteins were extracted from SWISSPROT (version 50.3) by European Bioinformatics Institute, Hinxton Cambridge, United Kingdom using the same rules as CL317.

### 3.2. Generalized Chaos Game Representation (GCGR) of Protein Primary Sequences

The chaos game representation (CGR) was initially introduced to visualize DNA sequences [29] and later for protein sequences as well [30]. Here, we further developed a generalized chaos game representation (GCGR) to represent a protein sequence by a 2-dimensional numerical feature vector describing the frequency of 20 amino acids and their neighbor information in the sequence. The construction of GCGR consists of three steps:

#### 3.2.1. Step 1: Convert a Protein Sequence into a Sequence on an Alphabet of Size 6

We converted the 20 amino acids into six groups (Table 5). Specifically, Proline (P), Glycine (G) and Cysteine (C) formed three separate groups because of their unique backbone properties. The remaining 17 amino acids were classified into the other three groups according to their hydropathy scale including strongly hydrophilic (denoted by H), strongly hydrophobic (L), and weakly hydrophilic or weakly hydrophobic (S) [31]. As a result, each primary protein sequence could be uniquely represented by a string on the alphabet {H, L, S, P, G, C}. For example, the protein sequence “YAMQESHFTCI” can be represented by “SLLHHSHLSCL” according to Table 5.

#### 3.2.2. Step 2: Construct the GCCR Plot

**Figure 2:**
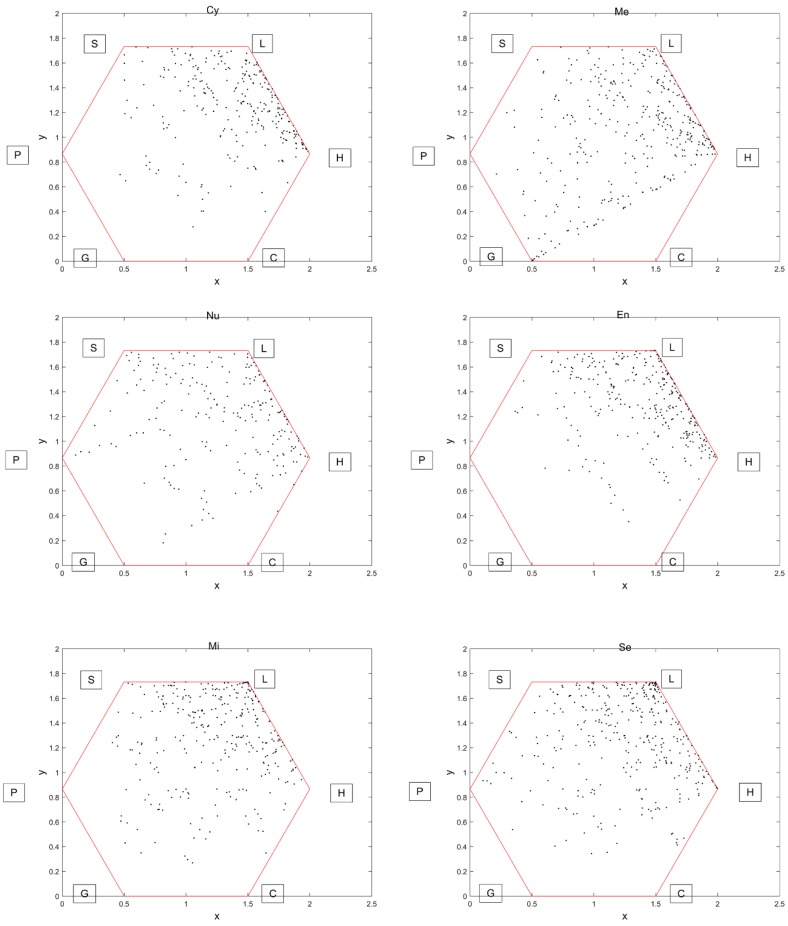
The GCGRs of primary sequence for proteins from six subcellular locations GCGR: Generalized Chaos Game Representation.

Firstly, we drew a regular hexagon, in which each vertex is associated with a distinct label of H, L, S, P, G and C, and each edge is of unit length. Then, for each encoded primary sequence in the first step, we plotted its letters sequentially as vertices inside the hexagon as follows: the first vertex, corresponding to the first letter of the primary sequence, was placed in the center of the hexagon; and the i-th vertex, corresponding to the i-th letter, was placed in the middle of the first (i-1)-th vertices and the vertex representing the i-th letter in the hexagon. After that, a plot named the GCGR of the primary sequence was drawn. As examples, we plotted in Figure 2 the GCGRs for six representative proteins with each belonging to a different subcellular location. From the six GCGR figures, we can directly retrieve some valuable information: for proteins in the Cy and Nu classes, the plotted points are close to vertices H and L; the protein in the Me class are uniformly distributed around all the vertices except for C; proteins in the Nu and En classes have fewer points around vertices G, C and P, G, respectively; proteins in the last two classes are almost uniformly distributed. In a word, the proteins in different subcellular locations distributed differently in the GCGR plots.

#### 3.2.3. Step 3: Convert Each Protein Sequence into a 2-D Vector according to its GCGR Plot

**Figure 3:**
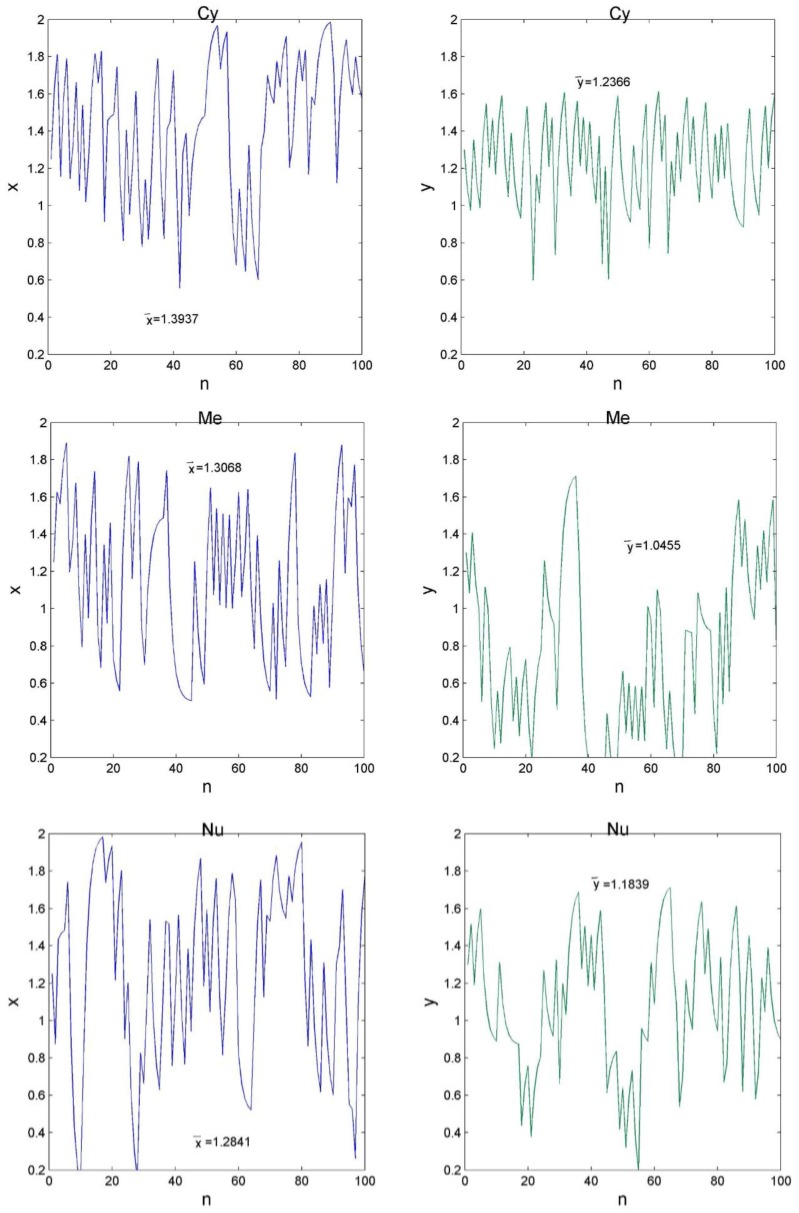
Six time series that represent the first three GCGRs in Figure 2. Each panel in Figure 2 gives rise to two time series.

**Figure 4:**
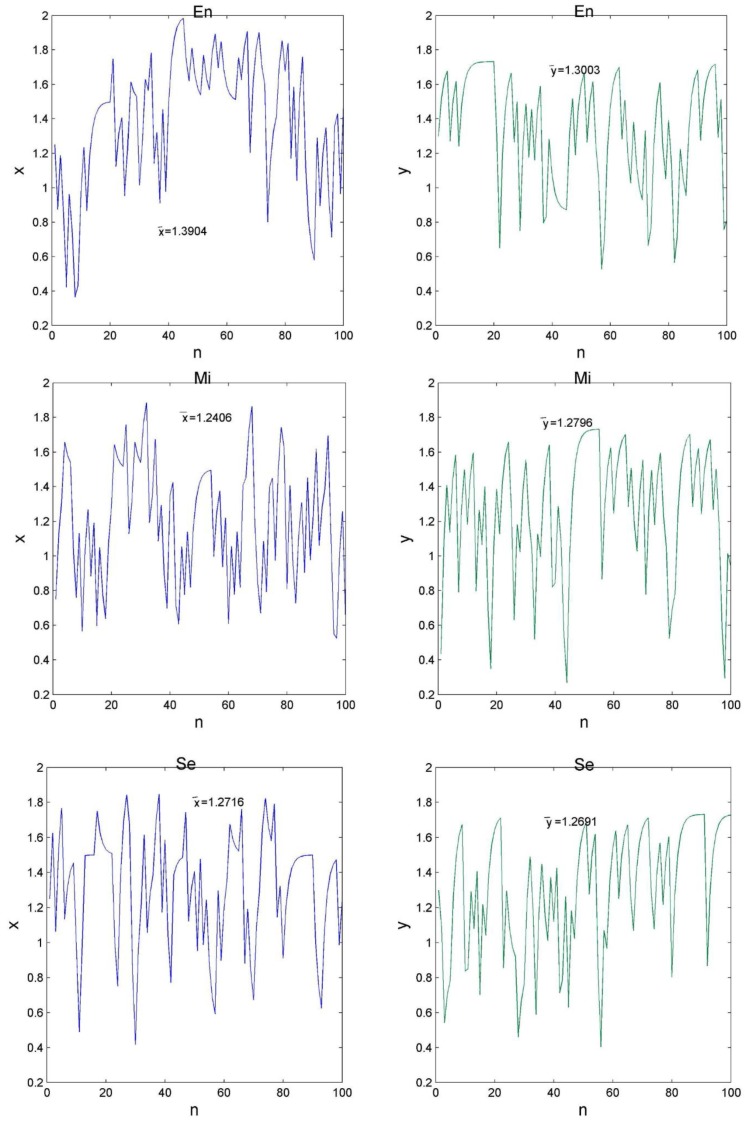
Six time series that represent the last three GCGRs in Figure 2. Each panel in Figure 2 gives rise to two time series.

As can be seen from Figure 2, each letter in the protein sequence corresponds to a (x, y)-coordinate in the GCGR plot. We then modelled the GCGR plot as a combination of two series: one is composed of the x-coordinates and the other is composed of the y-coordinates, which were named x-series and y-series, respectively. As can be seen from Figure 3 and Figure 4, there are many useful observations: (1) The average values of the x-series and y-series for proteins in the En-class, denoted as $\bar{x}$ and $\bar{y}$ respectively, tend to be greater than those for proteins in the other classes; (2) Proteins in the first class Cy also have a large $\bar{x}$, but do not have a large $\bar{y}$; (3) Proteins in the last two classes Mi and Se have moderate $\bar{x}$ and $\bar{y}$, respectively. 

**Figure 5:**
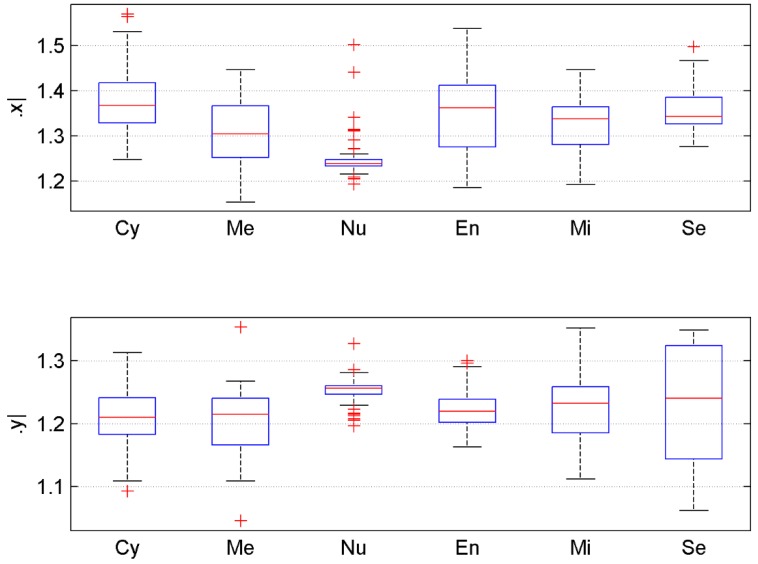
The boxplots for the $\bar{x}$ and $\bar{y}$ of all the proteins in dataset CL317 grouped into the six subcellular locations.

However, unlike proteins in the Se class, those in the Mi class have a greater $\bar{y}$ than its $\bar{x}$; (4) $\bar{y}$ of proteins in the second class Me is the smallest among all classes. In summary, $\bar{x}$ and $\bar{y}$ are two effective numerical features to identify subcellular location of proteins. It is not surprising since$\bar{x}$ and $\bar{y}$ contain not only the information about amino acids frequencies, but also their order in a protein sequence. For a better view, we also drew in Figure 5 the boxplots of $\bar{x}$ and $\bar{y}$ for proteins in each of the six classes in CL317. 

Theoretically, a class with narrow variation scope and less outliers can be discriminated more robustly. As can be seen, the proteins in the Nu class have substantially narrower variation with $\bar{x}$ ranges approximately from 1.22 to 1.26 and $\bar{y}$ ranges approximately from 1.23 to 1.28. For En, though $\bar{x}$ is widely distributed, $\bar{y}$ is more centralized, which can be used to differentiate this class. Similarly for Se, though $\bar{y}$ is widely distributed, $\bar{x}$ is more centralized. Finally, it is of note that all six classes has different medians and relatively differentiable variation scopes for both $\bar{x}$ and $\bar{y}$. Therefore, by combining $\bar{x}$ and the $\bar{y}$, it is possible to predict the localization of most proteins.

### 3.3. Novel Statistics and Information Theory (NSI) of Protein Primary Sequences

In order to acquire more position information of the primary sequence, we presented a novel statistics and information theory based method to extract features from the protein primary sequence. Different from the previous section, we just classified the 20 amino acids into three groups in view of their hydropathy profiles [21]: (1) internal group, in which the residues tend to appear in the inner side of the protein spatial structure, (2) external group, in which the residues tend to occur at the surface, and (3) ambivalent group, in which the residues do not have fixed common positions. Then, a protein sequence can be transformed into a 3-letter string according to the following rule:

(1) $$F(P(j))=\left\{ \begin{array}{l} F\;if\;P(j)=F,I,L,M,V\\ D\;if\;P(j)=D,E,H,K,N,Q,R\\ S\;if\;P(j)=S,T,Y,C,W,G,P,A \end{array}\right.$$

where P(j) represents the jth letter in the protein primary sequence P, and F(P(j)) presents the encoded letter for P(j). For example, given a protein sequence P=YAMQESHFTCI, its encoded sequence is F(P)=SSFDDSDFSSF.

After that, we calculated the position features of the encoded sequence to represent its local information. Specifically, let W(k)(k∈{F,D,S}) be the position sequence of a given amino acid k. We calculated the intervals between two consequent positions in W(k), which formed a new numerical distance sequence denoted by N(k). Obviously, N(k) contains the positional and distribution information of the given amino acid k in the primary sequences. For instance, for the encoded amino acid sequence, the position sequences for the reduced amino acids F, D, and S are: W(F)=(3,8,11), W(D)=(4,5,7), W(S)=(1,2,6,9,10). Then the symbolic sequences are cyclic and their numerical sequences N(k) are: N(F) = (5,3), N(D) = (1,2), N(S) = (1,4,3,1). The numerical sequence N(k) provides a new profile to characterize correlation residues of the given sequence. In fact, the interval distance between two occurrences of k can be denoted by a random variable x. We calculated the probability $p_{k}(x)$ of the Matthew’s correlation coefficient (MCC) of the variable x and obtain its distribution function. Based on the probability theory, we can further calculate the mean value $E_{k}(x)$ and the variance $D_{k}(x)$ by:

(2) $$E_{\left(k\right)}(x)=\sum_{x}x\times p_{\left(k\right)}(x)$$

(3) $$D_{\left(k\right)}(x)=E_{\left(k\right)}(x^{2})-{\left[E_{\left(k\right)}(x)\right]}^{2}$$

Then, we defined the positional information $I_{\left(k\right)}(x)$ as follows:

(4) $$I_{\left(k\right)}(x)=E_{\left(k\right)}(x)/\sqrt{D_{\left(k\right)}(x)}$$

where $I_{\left(k\right)}(x)$ is a pivotal statistic for comparing the degree of variation from one data to another. Finally, $I_{\left(k\right)}(x)(k\in \{F,D,S\})$ appropriately characterize the positional information of three encoding letters and thus form a novel feature vector of a protein primary sequence.

### 3.4. Unitary Distance

In this article, rather than the serial combination method which combines different feature vectors into a super-vector, the parallel combination method combines two feature vectors by a complex vector [32], which was defined by

(5) z=u+vi

where *i* is an imaginary unit. Here, we defined the parallel combined feature space on ℜ as C={u+vi|u∈A,v∈B}. Thus C is an m-dimensional complex vector space, where m=max(dimA,dimB). The inner product of two vectors in the complex space is given by $(a,b)=a^{H}b$, where a,b∈C, and H is the denotation of conjugate transpose. The complex vector space defined by the above inner product is usually called unitary space. The norm in unitary space is given by:

(6) $$|z||=\sqrt{z^{H}z}=\sqrt{\sum_{k=1}^{n}\left(a_{k}^{2}+b_{k}^{2}\right)}$$

where $z={\left(a_{1}+i\cdot b_{1},\cdots ,a_{n}+i\cdot b_{n}\right)}^{T}$.

Then, the unitary distance between two complex vectors $z_{1}$ and $z_{2}$ is calculated by:

(7) $$|z_{1}-z_{2}||=\sqrt{{\left(z_{1}-z_{2}\right)}^{H}(z_{1}-z_{2})}$$

### 3.5. Performance Assessment

For evaluating the effectiveness of two proposed features GCGR and NSI, we first introduced an easy model for fast predicting protein subcellular location, which is described as follows: for a given protein, its numeric features from GCGR and NSI were firstly extracted. Then, these two features are concatenated in parallel and then classified by a classifier free prediction model. As well-acknowledged, all the prediction models in the real number space could be extended to the complex number space by using different similarity measures. For example, the Euclidian distance is a commonly-used similarity metric in the real space, while the unitary distance is often used in the complex space, which was adopted in this paper. Note that the dimensionalities of u and v in the complex space must be equal, pad the lower-dimensional one with $\bar{x}/\bar{y}$ until its dimensionality is equal to the higher-dimensional one before vectors combination.

In order to measure the predictive capability of the algorithm, we adopted the following commonly used measures:

(8) $$\mathrm{Sensitivity}(S_{n})=TP/\left(TP+FN\right)$$

(9) $$\mathrm{Specificity}(S_{p})=TN/\left(TN+FP\right)$$

(10) $$\mathrm{MCC}=\frac{\left(TP\times TN)-(FP\times FN\right)}{\sqrt{\left(TP+FP)\times (TP+FN)\times (TN+FN)\times (TN+FP\right)}}$$

(11) $$\mathrm{Overall}\;\mathrm{prediction}\;\mathrm{accuracy}(A_{c})=\frac{\sum_{i}TP_{i}}{N}\;(i=1,2,3,\ldots n)$$

where True Positive (TP) represents the number of true positives in its subcellular location, True Negative (TN) represents the number of true negatives in its subcellular location, False Positive (FP) denotes the number of false positives and False Negative (FN) denotes the number of false negatives in its subcellular location. N is the total number of the protein sequences. Sensitivity means the rate of correct prediction. Specificity means the reliability level for predictive model. The Matthew’s correlation coefficient (MCC) shows the comprehensive performance of the prediction algorithm. 

In this paper, all the experiments were completed by MatLab and a Library for Support Vector Machines (LIBSVM). In the experiments, we chose the jackknife test for validating the performance of each model. The jackknife test is done by dropping in turn each sample from the data set as the test sample and fitting the model for the remaining set of observations as the training samples. The predicting accuracy can be obtained by the right classified samples divided by the total number of samples. It worth’s noticing that though there is no parameter in our feature construction process, there are two model parameters for LIBSVM, namely c and g. We adopted a grid search to find the best c and g in the jackknife test. Specifically, c and g both varied from 2^−5^ to 2^5^ with a multiple 2, and the best c is 8 and g is 0.0625 for the dataset CL317, while the numbers are 16 and 0.125 respectively for the dataset ZW225.

## 4. Conclusions

In this study, we first proposed a novel and quick method for predicting yeast subcellular locations based on generalized chaos game representation of the protein primary sequence and the statistics and information theory to uncover the residues distribution among the sequence. Implementation on two benchmark yeast datasets suggests that this model achieves comparable classification performance as those of machine learning-based classifiers. In addition, a fusion model incorporating GCGR and NSI with some known features including PwAAC and Dipeptide were presented, which gains the highest overall accuracy and MCC on the two benchmark datasets. The results also indicate that the new features extracted contain some useful information, which is not mined in previous methods.

## Figures and Tables

**Table 1:** The prediction results of dataset CL317 using unitary distance based on GCGR + NSI features in the jackknife test.

	Cy	Me	Nu	En	Mi	Se
Sn (%)	91.8	85.3	86.5	93.7	83.3	74.5
Sp (%)	86.2	99.6	91.9	86.2	91.5	90.9
MCC	0.83	0.83	0.86	0.88	0.86	0.81
Acc	0.8825					

**Table 2:** The prediction results of dataset ZW225 using unitary distance based on GCGR + NSI features in the jackknife test.

	Me	Cy	Nu	Mi
Sn (%)	0.6617	0.8286	0.88	0.8439
Sp (%)	0.7792	0.7432	0.9383	0.8841
MCC	0.6863	0.6789	0.9115	0.7745
Acc	0.7736			

**Table 3:** Comparison of prediction performance for CL317 in the jackknife test.

Predictor	MCC						Acc
	Cy	Me	Nu	En	Mi	Se	
[15]	0.80	0.77	0.73	0.90	0.74	0.68	0.827
[23]	0.87	0.90	0.86	0.95	0.86	0.80	0.909
[24]	0.84	0.85	0.84	0.91	0.77	0.80	0.88
[25]	0.89	0.88	0.87	0.95	0.88	0.78	0.911
[6]	0.946	0.909	0.885	0.957	0.882	0.706	0.912
This paper	0.896	0.913	0.929	0.892	0.853	0.905	0.921

**Table 4:** Comparison of prediction performance for ZW225 in the jackknife test.

Predictor	MCC				Acc
	Me	Cy	Nu	Mi	
[3]	0.933	0.90	0.634	0.60	0.831
[15]	0.91	0.929	0.732	0.68	0.858
[24]	0.921	0.871	0.732	0.64	0.84
[6]	0.91	0.871	0.756	0.72	0.849
This paper	0.909	0.892	0.867	0.778	0.889

**Table 5:** The six classes of the 20 amino acids.

Classification	Abbreviation	Amino Acids
Strongly hydrophilic or polar	H	H, R, D, E, N, Q, K
Strongly hydrophobic	L	L, I, V, A, M, F
Weakly hydrophilic or weakly hydrophobic (ambiguous)	S	S, T, Y, W
Proline	P	P
Glycine	G	G
Cysteine	C	C
